# The PTB-Associated Splicing Factor/Peroxisome Proliferator-Activated Receptor Gamma Axis Regulates Autophagosome Formation in Human Pancreatic Cancer Cells

**DOI:** 10.1089/biores.2015.0018

**Published:** 2015-07-01

**Authors:** Tamotsu Tsukahara, Hisao Haniu, Yoshikazu Matsuda

**Affiliations:** ^1^ Department of Molecular Pharmacology and Neuroscience, Nagasaki University Graduate School of Biomedical Sciences, Nagasaki, Japan.; ^2^ Institue for Biomedical Sciences, Shinshu University Interdisciplinary Cluster for Cutting Edge Research, Matsumoto, Nagano, Japan.; ^3^ Clinical Pharmacology Educational Center, Nihon Pharmaceutical University, Ina-machi, Saitama, Japan.

**Keywords:** autophagy, cell proliferation, LC3B, p62, pancreatic cancer cell, peroxisome proliferator-activated receptor gamma, PTB-associated splicing factor

## Abstract

Peroxisome proliferator-activated receptor gamma (PPARγ) is a nuclear receptor that plays a major regulatory role in metabolic function. It is overexpressed in many types of cancer cells, suggesting that regulation of PPARγ may also affect carcinogenesis. Our previous study suggested that PTB-associated splicing factor (PSF) is a PPARγ-interacting protein and growth regulator of colon cancer cells. In addition, PSF has been shown to be involved in several important regulatory steps of cancer cell proliferation. In this study, we aimed to investigate the relationships between PSF and PPARγ in pancreatic cancer by evaluating the effects of PSF expression in pancreatic cancer cell lines. PSF expression affected the expression of PPARγ, and knockdown of PSF using specific small-interfering RNA (siRNA) significantly suppressed the proliferation of pancreatic cancer cells. Furthermore, PSF knockdown induced cell growth inhibition and autophagosome formation through inhibition of PPARγ. Interestingly, Panc-1 cells were more susceptible to PSF knockdown-induced autophagy than MIA-PaCa-2 cells. Thus, our data indicated that PSF was an important regulator of autophagy and played critical roles in the survival and growth of pancreatic cancer cells. The PSF-PPARγ axis may play a role in the control of pancreatic cancer pathogenesis. This study is the first to describe the effects of PSF on pancreatic cancer cell growth and autophagy associated with PPARγ.

## Introduction

Pancreatic cancers are associated with a poor prognosis, with 1- and 5-year survival rates of 20% and less than 5%, respectively; however, the mechanisms underlying the aggressiveness of pancreatic cancer are not fully understood.^1^ Pancreatic cancer is usually diagnosed at an advanced stage, and no effective therapies are available owing to both the aggressiveness of pancreatic tumors and the relatively poor sensitivity of these tumors to chemotherapy or radiation therapy.^2^

PTB-associated splicing factor (PSF) is a nuclear protein involved in transcription regulation, pre-mRNA splicing, and DNA repair.^3–5^ PSF is a putative tumor-suppressor protein that contains an RNA-binding domain and a DNA-binding domain (DBD).^6^ The DBD binds and represses transcription of target genes that have a PSF-binding site. Thus, PSF is an extremely complex protein that may be a critical component involved in the transcriptional repression of many different genes through various mechanisms.

Interestingly, changes in the expression of PSF have been shown to be associated with cancer development and progression.^7^ For example, Hayes et al. reported the potential for targeting the mRNA splicing machinery, including PSF, as a treatment strategy for pancreatic carcinomas.^8^ Moreover, Wang et al. reported that PSF has a central role in the reversible regulation of cell proliferation and tumorigenesis.^9^ In a recent study, we showed that PSF expression blocked the proliferation of cancer cells expressing low levels of peroxisome proliferator-activated receptor gamma (PPARγ), but not in cancer cells expressing high levels of PPARγ.^5^ Furthermore, the PPARγ-PSF complex has been shown to induce the downregulation of the autophagy-related protein LC3B in colon cancer cells,^10^ thus suggesting that the PSF-LC3B axis may function as a potential endogenous modulator of cancers. We speculated that PSF might interact with PPARγ, a nuclear receptor involved in cell proliferation and apoptosis. Knockdown of PSF in PPARγ-expressing DLD-1 colon cancer cell lines results in loss of the autophagic marker LC3B and a corresponding induction of apoptosis through caspase-3. Interestingly, the same consequence of PSF knockdown is not observed in PPARγ-low HT-29 cells. Taken together, these studies suggest that PSF is a regulator of cell death in some cancer cells and that the relative expression level of PPARγ appears to play an important role in mediating cell death.

Therefore, from these studies, PSF and PPARγ appear to be two attractive targets for the development of novel cancer treatments. In this study, we aimed to determine the correlation between PSF and PPARγ protein levels in pancreatic cancer cells and to examine the effects of different PSF/PPARγ expression levels on cell proliferation using human pancreatic cancer cell lines expressing varying levels of PSF. Our data give important insights into the molecular mechanisms of pancreatic carcinogenesis.

## Materials and Methods

### Materials

Rabbit polyclonal anti-LC3B antibodies (NB100-2220SS) were purchased from Novus Biologicals. Mouse monoclonal anti-β-actin antibodies (sc-47778) and anti-PPARγ antibodies (E-8; sc-7273) were purchased from Santa Cruz Biotechnology, Inc. Rabbit polyclonal anti-p62 antibodies (PM045) were purchased from Medical & Biological Laboratories. A Premo Autophagy Sensor (LC3B-FP) was purchased from Molecular Probes.

### Cells and cell culture

The human pancreatic cancer cells lines MIA-PaCa-2 and Panc-1 were gifts from the RIKEN Cell Bank. Cells were cultured in Dulbecco's modified Eagle's medium (DMEM; Nacalai) supplemented with 10% fetal bovine serum (FBS). Cultures were incubated in a humidified atmosphere of 95% air and 5% CO_2_ at 37°C.

### Protein extraction and western blot analysis

MIA-PaCa-2 and Panc-1 cells were washed twice with ice-cold phosphate-buffered saline and solubilized in the RIPA buffer (ATTO). Clarify the cell lysate by centrifugation at 14,000 *g* for 10 min to pellet the cell debris, and the protein in the supernatant was quantified using a Protein Quantification Kit-Rapid (Dojindo). An equivalent amount of protein from each sample was subjected on 5–20% Mini-PROTEAN TGX Precast Gels (Bio-Rad) and transferred to Trans-Blot Turbo Mini PVDF Transfer Packs. The membranes were blocked in 5% Block Ace (DS Parma Biomedical Co. Ltd.) for 1 h and then incubated with a primary antibody in TBS-T with 5% Block Ace for 12 h at 4°C. Bands were visualized with EzWestLumi plus (ATTO).

### Measurement of cell proliferation

Cells were seeded into the wells of the plate at densities of 1×10^4^ cells in 100 μL of cell culture media, and proliferation rates were determined using a Cell Counting Kit-8 (Dojindo). After cells were incubated for 24 h, 10 μL of the Cell Counting Kit-8 solution was added to each well, and the plates were incubated for 1 h in an incubator at 37°C with 5% CO_2_. The amount of formazan dye was determined by measuring the absorbance at 450 nm in a microplate reader (Awareness Technology).

### Quantitative real-time polymerase chain reaction

Total RNA from cultured MIA-PaCa-2 and Panc-1 cells was extracted using a NucleoSpin RNA II kit (TaKaRa) according to the manufacturer's protocol. Total RNA (0.5 μg) was used for the subsequent synthesis of cDNA with a ReverTra Ace qPCR RT Kit (Toyobo), as recommended by the manufacturer. The levels of mRNA were measured using an ECO Real-Time PCR system (Illumina, Inc.) and SYBR Green Real-Time PCR Master Mix-Plus (Toyobo) with the following primer pairs: PPARγ, 5′-GTGGCCGCAGA TTTGAAAGAAG-3′ (forward) and 5′-TGTCAACCA TGGTCATTTCG-3′ (reverse); PSF, 5′-ACGGTCAT TCCGTATGCAGC-3′ (forward) and 5′-GGATAGC CCCCATGACGAT-3′ (reverse); and β-actin, 5′-AGG CACCAGGGCGTGAT-3′ (forward) and 5′-GCCCAC ATAGGAATCCTTCTGAC-3′ (reverse). The polymerase chain reaction (PCR) product specifically was verified by a melting curve analysis. Levels of PPARγ and PSF expression were normalized to the endogenous reference gene β-actin using the relative quantitative method (ΔΔCt), as previously reported.^5,10^

### siRNA construction and transfection

The expression of PSF in Panc-1 cells was inhibited by transfection with small interfering RNAs (siRNAs) targeting PSF (Santa Cruz Biotechnology) using Lipofectamine RNAiMAX (Invitrogen), as previously reported.^5,10^ Cells were cultured in 6-well plates (Iwaki) at a density of 5×10^4^ cells/well in DMEM containing 10% FBS. Cells were then transfected with 100 pmol/mL of mRNA-specific siRNAs or a scrambled control siRNA. The reduction in PSF levels was confirmed using western blotting analysis.

### Reporter gene assays

PPARγ activation was measured in Panc-1 and MIA PaCa-2 cells transfected with 125 ng of the pGL3-PPRE-acyl-CoA oxidase luciferase vector, 62.5 ng of the pcDNA3.1-PPARγ vector, and 12.5 ng of the pSV-β-galactosidase vector (Promega), constructed as previously reported.^11,12^ At 24 h after transfection, cells were treated with Opti-MEM (Invitrogen) containing the test compounds dissolved in DMSO (up to 0.1%) and cultured for an additional 20 h. The luciferase activity was measured using the ONE-Glo Luciferase Assay System (Promega) and a LuMate microplate luminometer (Awareness Technology, Inc.).

### Autophagy detection

The induction of autophagy was detected with a Premo Autophagy Sensor LC3B-GFP BacMam 2.0 kit (Invitrogen) as previously reported.^10^ Briefly, 1 day after siRNA treatment, Panc-1 and MIA PaCa-2 cells were transduced with BacMam LC3B-GFP. Chloroquine diphosphate (100 μM) was used to induce autophagy (positive control).

### Statistical analysis

Student's *t*-tests were used for statistical comparisons. Differences were considered significant when the *p*-value was below 0.05.

## Results and Discussion

**FIG. 1.**
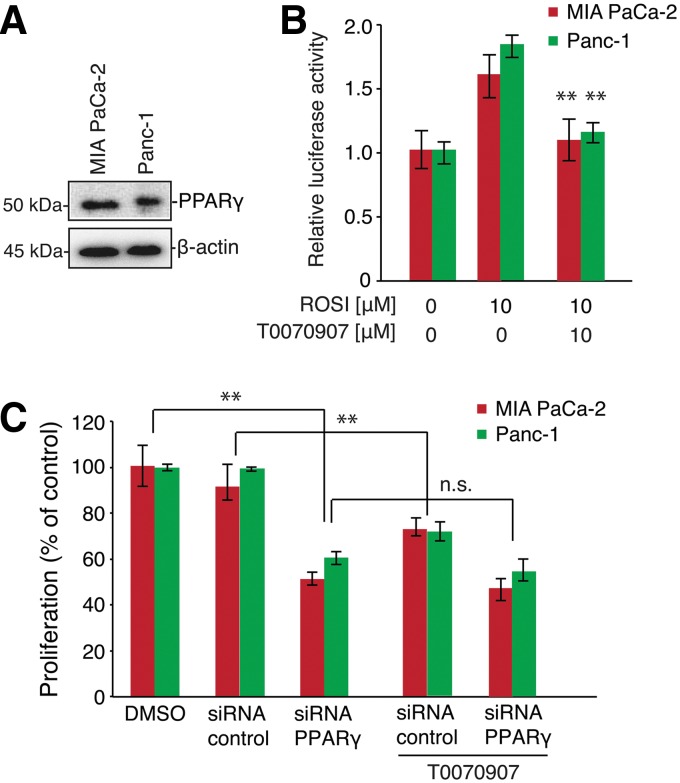
Comparison of endogenous peroxisome proliferator-activated receptor gamma (PPARγ) expression. **(A)** Whole cell extracts were prepared from cells and analyzed by immunoblotting using anti-human PPARγ antibodies. β-Actin was used as loading control. **(B)** Effects of PPARγ ligands on reporter activation and inhibition in MIA PaCa-2 and Panc-1 cells. Cells were transiently transfected with a pGL3-PPRE-acyl-CoA oxidase luciferase reporter vector. The cells were treated with 10 μM rosiglitazone with or without synthetic PPARγ antagonist (T0070907) for 20 h. Luciferase activity was normalized to Renilla luciferase activity. Data are expressed as mean±SEM (*n*=4); **p<0.01. **(C)** PPARγ knockdown inhibited cell growth in MIA PaCa-2 and Panc-1 cells. Cells (1×10^4^ cells/well) were seeded in 96-well plates after transfection and incubated for 48 h at 37°C with 5% CO_2_. Next, cells were incubated with T0070907 for 48 h at 37°C, and 10 μL of Cell Counting Kit-8 solution was added to the medium. After incubating for 1 h, the amount of orange formazan dye generated was determined by measuring the absorbance at 450 nm using a microplate reader. Data are expressed as mean±SEM (*n*=4); **p<0.01.

First, we determined the protein levels of PPARγ in MIA PaCa-2 and Panc-1 cells. Both cell lines expressed PPARγ (Fig. 1A). In addition, we analyzed the activity of PPARγ in both cell lines by luciferase reporter assays. In cells treated with 10 μM rosiglitazone, an agonist for PPARγ, for 24 h, MIA PaCa-2 and Panc-1 cells exhibited a 1.8- and 1.6-fold higher PPARγ activity than that in vehicle (DMSO)-treated cells. Interestingly, treatment with the synthetic PPARγ inhibitor, T0070907, blocked rosiglitazone-dependent PPARγ activation. These results suggested that rosiglitazone could activate PPARγ in both cell lines (Fig. 1B). Moreover, knockdown of PPARγ also decreased cell proliferation in both cell lines, and T0070907 did not affect cell proliferation in PPARγ knockdown cells. These results suggest that PPARγ was an important regulator of cell proliferation in pancreatic cancer cells.

**FIG. 2.**
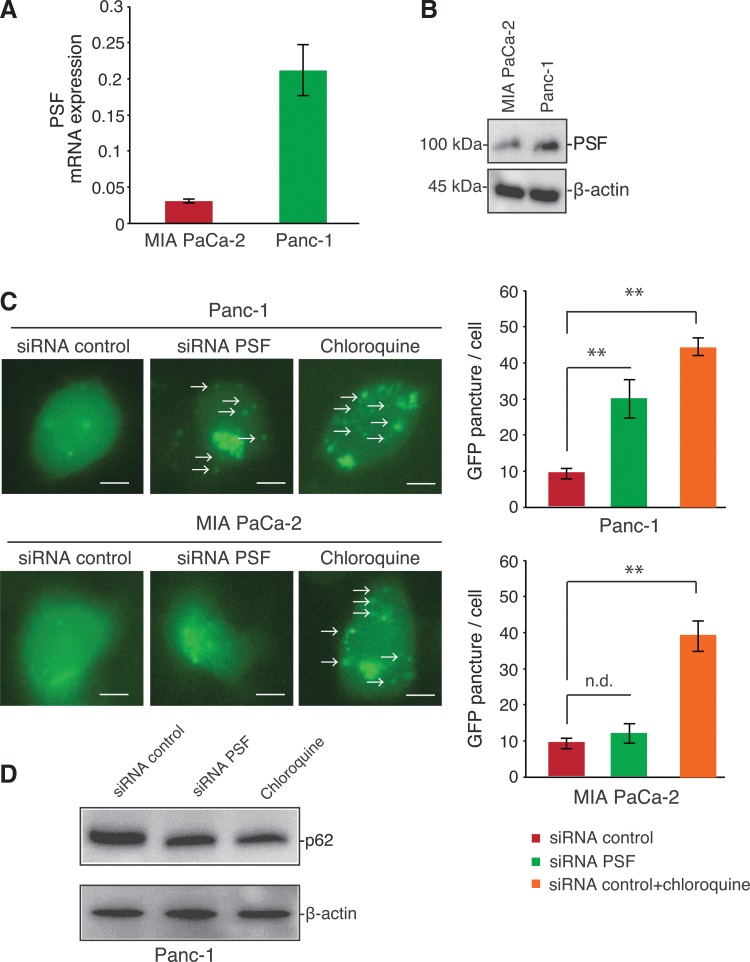
PTB-associated splicing factor (PSF) knockdown induced LC3B in Panc-1 cells, but not MIA PaCa-2 cells. **(A)** Expression of PSF in MIA PaCa-2 and Panc-1 cells. Real-time PCR measurement of *PSF* mRNA expression in MIA PaCa-2 and Panc-1 cells. PSF expression was normalized to β-actin expression. Data are expressed as the mean±SEM (*n*=3); **p<0.01. **(B)** Total protein was extracted from MIA PaCa-2 and Panc-1 cells. Whole-cell lysates were subjected to western blot analysis to determine the levels of PSF expression. β-Actin was used as a protein-loading control. **(C**, left panel**)** Autophagosome detection with a Premo Autophagy Sensor. MIA PaCa-2 and Panc-1 cells transfected with PSF small-interfering RNA (siRNA) were plated at a density of 5×10^3^ cells/well. Cells were then transduced with LC3B-GFP. Twenty-four hours later, the cells were analyzed by quantifying the florescence from vesicular structures **(C**, right panel; indicated by arrows**)** in the perinuclear region. Scale bar, 10 μm. **(D)** Western blot analysis of p62/SQSTM1 protein after PSF knockdown. Panc-1 cells transfected with PSF siRNA were plated at a density of 1×10^5^ cells/well and subjected to western blot analysis to determine p62/SQSTM1expression levels. β-Actin was used as a protein loading control. Chloroquine was used as a positive control.

As shown in Figure 2A and B, we next investigated the expression of PSF mRNA and protein in pancreatic cancer cells using quantitative real-time PCR and western blotting. Panc-1 cells exhibited high expression of PSF, while MIA PaCa-2 cells exhibited a relatively lower expression of PSF.

Our previous data suggested that PSF markedly decreased expression of the autophagic molecule LC3B,^10^ which localizes to the accumulated autophagic vacuoles in the cytoplasm of cells undergoing autophagy.^13^ Therefore, we also examined the effects of PSF knockdown on LC3B expression and localization. As shown in Figure 2C, the localization of GFP-LC3B was significantly increased in vesicular structures in the cytosolic region in Panc-1 cells, which expressed a high level of endogenous PSF. In contrast, little fluorescence representing LC3B expression was observed in MIA PaCa-2 cells, which express a lower level of PSF. Furthermore, p62/SQSTM1 has been suggested to be specifically degraded by autophagy, with decreases in expression observed in response to activation of autophagy.^14^ Hence, we determined the effects of PSF knockdown on p62/SQSTM1 protein levels in Panc-1 cells. As shown in Figure 2D, the expression levels of p62/SQSTM1 protein were decreased in Panc-1 cells after PSF knockdown. These data demonstrated that PSF expression was critical for autophagosome induction.

**FIG. 3.**
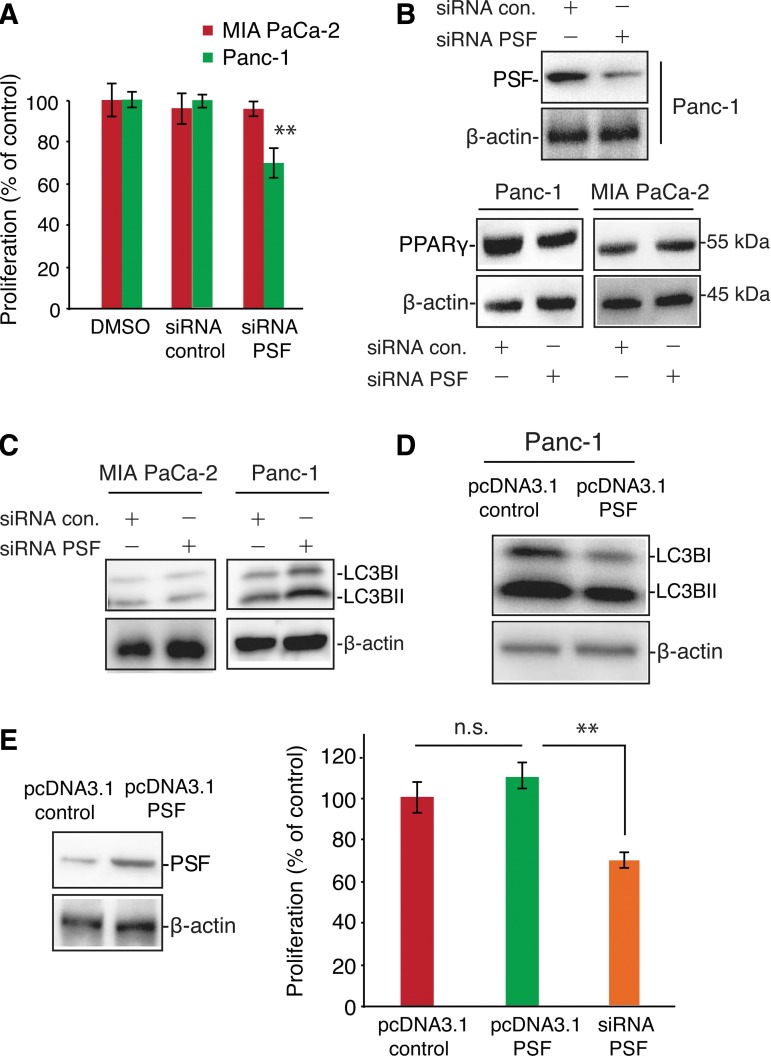
Downregulation of PSF inhibits the proliferation of Panc-1 cells. **(A)** After transfection with siRNA control or PSF siRNA, cells were replated in 96-well plates (1×10^4^ cells/well) and cell growth inhibition was measured using a Cell Counting Kit-8 after 24 h. Data are expressed as mean±SEM (*n*=4); **p<0.01. **(B)** MIA PaCa-2 and Panc-1 cells transfected with siRNA control or PSF siRNA were subjected to western blot analysis for detection of PSF and PPARγ expression. β-Actin was used as a protein-loading control. **(C)** PSF knockdown induced LC3B expression in Panc-1 cells. MIA PaCa-2 and Panc-1 cells transfected with siRNA control or PSF siRNA were subjected to western blot analysis for detection of LC3B expression. β-Actin was used as a protein-loading control. **(D)** PSF overexpression decreased LC3B protein levels in Panc-1 cells. MIA PaCa-2 and Panc-1 cells were transfected with a pcDNA3.1 empty vector or vector containing PSF and subjected to western blot analysis for determination of LC3B expression levels. β-Actin was used as a protein-loading control. **(E)** PSF overexpression restored cell proliferation in Panc-1 cells. Cells (1×10^4^ cells/well) were seeded in 96-well plates after transfection and treated with T0070907 for 48 h. Next, 10 μL of Cell Counting Kit-8 solution was added to the medium, and cells were incubated for an additional 1 h. The amount of orange formazan dye generated was determined by measuring the absorbance at 450 nm using a microplate reader. Data are expressed as mean±SEM (*n*=4); **p<0.01.

Next, to determine the role of PSF in regulating PPARγ expression, we examined the effects of PSF knockdown on PPARγ expression and cell proliferation in Panc-1 and MIA PaCa-2 cells. As shown in Figure 3A, PSF knockdown resulted in decreased cell proliferation in Panc-1 cells, but not MIA PaCa-2 cells. Moreover, after transfection with PSF siRNA, PPARγ expression levels were decreased in Panc-1 cells (Fig. 3B). Next, to determine the role of PSF in regulating LC3B expression, we examined the effects of PSF knockdown on LC3B expression in Panc-1 and MIA PaCa-2 cells. As shown in Figure 3C, after PSF knockdown, LC3B was increased in Panc-1 cells, but not in MIA PaCa-2 cells, which express a lower level of PSF. Consistent with our above data, the expression of LC3B was increased after transfection with PSF siRNA in Panc-1 cells. These inhibitory effects were reversed by PSF overexpression. Indeed, as shown in Figure 3D and E, PSF overexpression promoted cell proliferation in Panc-1 cells. Moreover, there was a correlation between the level of PSF expression and proliferation or autophagosome formation.

**FIG. 4.**
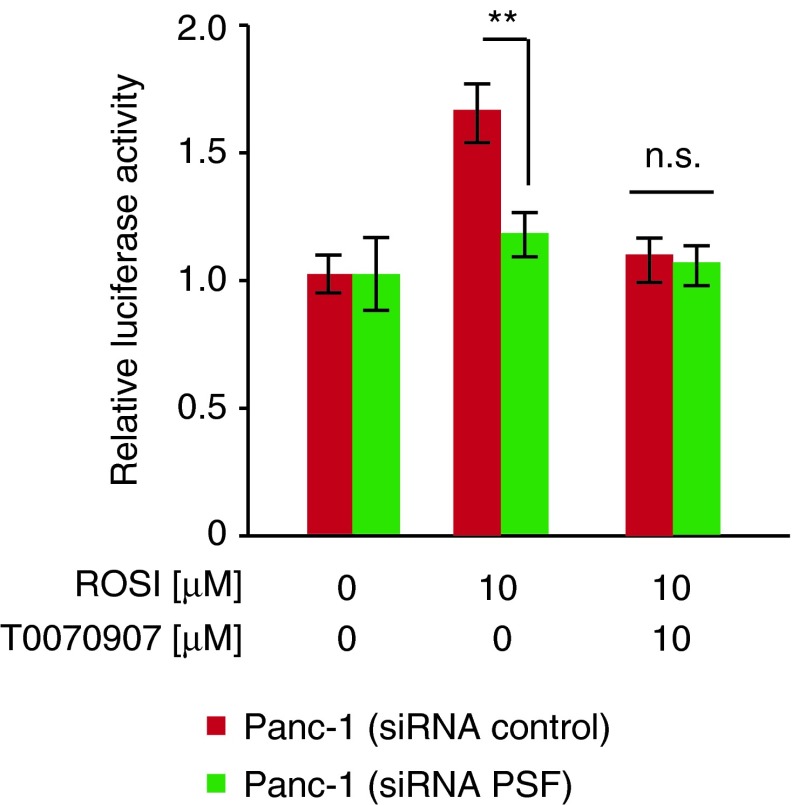
Comparison of PPARγ activation and inhibition. PPARγ activation by rosiglitazone (10 μM) and/or inhibition with T0070907 (10 μM) in Panc-1 cells transfected with siRNA control or PSF siRNA. Luciferase activity was normalized to Renilla luciferase activity. Data are expressed as mean±SEM (*n*=3); **p<0.01.

Our previous study suggested that inhibition of cell growth by the PPARγ antagonist is mediated by its inhibition of the PPARγ pathway.^12^ Finally, as shown in Figure 4, we could not detect the transcriptional activity of the PPRE-ACox-Luc reporter genes after PSF knockdown in Panc-1 cells. Taken together, our research provides important insights into the biological functions of the PSF-PPARγ axis and the autophagy-related protein LC3B and p62 improving our understanding of the various events in pancreatic cancer pathogenesis.

## Abbreviations Used

DBD: DNA-binding domain
DMEM: Dulbecco's modified Eagle's medium
FBS: fetal bovine serum
PPARγ: peroxisome proliferator-activator receptor gamma
PSF: PTB-associated splicing factor
siRNA: small-interfering RNA
TBS-T: Tris-buffered saline containing Tween 20
